# Reestablishment of Redox Homeostasis in the Nociceptive Primary Afferent as a Mechanism of Antinociception Promoted by Mesenchymal Stem/Stromal Cells in Oxaliplatin-Induced Chronic Peripheral Neuropathy

**DOI:** 10.1155/2021/8815206

**Published:** 2021-01-06

**Authors:** Anna Lethicia L. Oliveira, Gisele G. L. Santos, Renan F. Espirito-Santo, Gessica Sabrina A. Silva, Afrânio F. Evangelista, Daniela N. Silva, Milena B. P. Soares, Cristiane Flora Villarreal

**Affiliations:** ^1^Gonçalo Moniz Institute, Oswaldo Cruz Foundation, 40296-710, Brazil; ^2^College of Pharmacy, Federal University of Bahia, 40170-290, Brazil; ^3^SENAI Institute of Innovation in Advanced Health Systems (ISI SAS), University Center SENAI/CIMATEC, 41650-010, Brazil; ^4^National Institute of Science and Technology for Regenerative Medicine (INCT-REGENERA), Rio de Janeiro, RJ, Brazil

## Abstract

Painful neuropathy is a common adverse effect of oxaliplatin (OXL), a platinum-derivative chemotherapeutic agent. Oxidative stress and mitochondrial dysfunction are key factors contributing to the development of OXL-induced peripheral neuropathy (OIPN). Based on the antioxidant and antinociceptive properties of mesenchymal stem/stromal cells (MSC), the present study tested the hypothesis that MSC induce antinociceptive effects during OIPN by promoting regulation of redox environment and mitochondrial homeostasis in the nociceptive primary afferents. C57Bl/6 mice submitted to the OXL-chronic neuropathy induction protocol by repeated intravenous administration of OXL (1 mg/kg) were evaluated to determine the paw mechanical and thermal nociceptive thresholds using the von Frey filaments and cold plate tests, respectively. Two weeks after the neuropathy induction, mice were treated with bone marrow-derived MSC (1 × 10^6^), vehicle, or gabapentin (GBP, 70 mg/kg). Four weeks later, mitochondrial morphology, gene expression profile, and oxidative stress markers in the sciatic nerve and dorsal root ganglia (DRG) were evaluated by transmission electron microscopy, RT-qPCR, and biochemical assays, respectively. OXL-treated mice presented behavioral signs of sensory neuropathy, such as mechanical allodynia and thermal hyperalgesia. The behavioral painful neuropathy was completely reverted by a single administration of MSC, while the daily treatment with GBP induced only a short-lived antinociceptive effect. The ultrastructural analysis of the sciatic nerve and DRG of OIPN mice revealed a high proportion of atypical mitochondria in both myelinated and unmyelinated fibers. Importantly, this mitochondrial atypia was strongly reduced in MSC-treated neuropathic mice. Moreover, MSC-treated neuropathic mice showed upregulation of *Sod* and *Nrf2* mRNA in the sciatic nerve and DRG. In line with this result, MSC reduced markers of nitrosative stress and lipid peroxidation in the sciatic nerve and DRG from OIPN mice. Our data suggest that the reestablishment of redox homeostasis in the nociceptive primary afferents is a mechanism by which MSC transplantation reverts the OXL-induced chronic painful neuropathy.

## 1. Introduction

Sensory peripheral neuropathy is a common side effect seen in cancer patients treated with the chemotherapeutic agent oxaliplatin (OXL), and one of the main reasons for withdrawal of this antitumor therapy. OXL is a third-generation platinum analogue widely used for treatment of metastatic colon cancer, alone or in combination with other agents [1]. Structurally similar to cisplatin, OXL presents structural modifications that increases its antitumor activity and reduces its nephrotoxicity. On the other hand, OXL has a greater potential for neurotoxicity than other platinum compounds [2]. Approximately 20-50% of OXL-treated colorectal cancer patients develop peripheral neuropathy that persists for months to years after the end of chemotherapy [3]. Clinically, the symptoms of oxaliplatin-induced peripheral neuropathy (OIPN) include reduced pain threshold, paresthesia, and dysesthesia, affecting lower and upper extremities in a characteristic distribution of “glove and stocking” [3, 4]. Some clinical features are represented in animal models, which present a neuropathic syndrome induced by OXL also characterized by aberrant somatosensory processing and pain-like behaviors [5, 6].

Platinum concentrations in peripheral nervous tissue are similar to that found in the tumor tissue, and comparatively, much lower concentrations are found in the brain [7, 8]. At the cellular level, following intracellular hydrolysis, OXL accumulates in the neurons and forms platinum-DNA (Pt-DNA) adducts with both nuclear and mitochondrial DNA, resulting in DNA damage and inhibition of DNA replication and transcription, leading to the production of free radicals and neuronal apoptosis [9, 10].

The pathophysiological mechanisms of OIPN converge to three of the most extensively studied pathways: oxidative stress, inflammation, and apoptosis. Among them, oxidative stress associated with mitochondrial dysfunction in the sensory peripheral nerve has emerged as a central mechanism for induction and maintenance of the OIPN [11–14]. The mitotoxicity hypothesis considers that Pt-DNA adducts induce mitochondrial damage associated with depletion of energy production in nociceptive primary afferent neurons, resulting in axonal degeneration and abnormal spontaneous discharges. The accumulation of Pt-DNA adducts alters the expression pattern of mitochondrial matrix proteins and transmembranic and mitochondrial intermembranes, important for the oxidative phosphorylation activities. The phosphorylative machinery, which is also the primary locus of the peroxynitrite formation and reactions, presents the potential of the mitochondrial membrane to disintegrate due to the nitration of proteins. Consequently, disruption of phosphorylative activity exacerbates mitochondrial damage, which causes neuronal energy deficit [12, 15]. In fact, functional deficits in peripheral nerve mitochondria have been associated with the oxaliplatin-evoked painful neuropathy [16]. Oxidative stress induced by OXL can also cause mitochondrial dysfunction in neurons of the dorsal root ganglia (DRG), leading to cellular energy failure in nociceptive neurons [15].

One of the main problems concerning OIPN is related to its poor response to the currently available analgesic therapy. Clinical management of chemotherapy-induced neuropathic pain mainly includes gabapentinoids, such as gabapentin and pregabaline, and antidepressants, such as amitriptyline and duloxetine. However, despite being the first-line drugs for controlling neuropathic pain, their use is largely limited due to poor efficacy or undesirable side effects [4]. Moreover, none of these drugs has been shown to reverse cellular damage established after OXL cycles and restore organelle homeostasis in sensory neurons during chronic neuropathic states [4, 17]. Based on the role of mitochondrial damage to the OIPN development, pharmacological studies have investigated possible therapeutic effects of mitoprotective agents to prevent or relieve OXL-evoked pain. These include several antioxidant compounds, such as n-acetylcysteine, n-acetyl carnitine, glutathione, alpha-lipoic acid, and vitamin E, which, however, have shown limited efficacy due to failure in modifying redox signaling pathways [18].

Due to this gap in the pharmacological treatment, cell therapy with mesenchymal stem/stromal cells (MSC) has been investigated and considered a promising therapeutic approach for the treatment of sensory neuropathies. The strategy of mesenchymal cell transplantation for the treatment of chronic neuropathies is based on cell-induced analgesia and its potential for nervous system repair, which has been attributed to the neuroprotective, neuroregenerative, or immunomodulatory properties of these cells [19–21]. The therapeutic effect of bone marrow-derived MSC has been demonstrated in different types of experimental sensory neuropathies, such as diabetic neuropathy [22–24], traumatic nerve injury [25–27], spinal cord injury [28], and chemotherapy-induced neuropathy [19, 29–31].

MSC have an important antioxidant action, being capable of protecting tissues from the harmful effects of ROS [32–34], which may represent a therapeutic advantage for OIPN treatment. MSC induce the expression of antioxidant enzymes which constitute a crucial cellular protection system against oxidative stress [23, 35]. Additionally, the therapeutic potential of MSC in oxidative damage has also been attributed to mitochondrial transfer capacity between MSC and damaged tissue, leading to restoration of mitochondrial respiratory function [36–40]. The effects of MSC on cellular redox homeostasis may be due to the transference of soluble factors, which are also related to analgesia and neuroprotection [41–43], as well as mitochondrial donation [36]. These relevant properties of MSC confer an outstanding potential for repairing tissues that have undergone metabolic alterations and oxidative dysregulation, such as those observed during OIPN.

Considering this scenario, the present study was designed to evaluate the hypothesis that MSC transplantation reverts the oxaliplatin-induced sensory neuropathy via reducing oxidative stress and mitochondrial dysfunction in the nociceptive primary afferents.

## 2. Materials and Methods

### 2.1. Animals

Experiments were performed on male C57Bl/6 mice (20–23 g) obtained from the Animal Facilities of Instituto Gonçalo Moniz/FIOCRUZ (Brazil). Mice were housed under a 12 : 12 h dark-light cycle. Animals had access to water and food ad libitum and were housed in temperature-controlled rooms (22–25°C). All behavioral tests were performed between 8:00 a.m. and 5:00 p.m., and animals were only tested once. Animal care and handling procedures were in accordance with the National Institutes of Health guide for the care and use of laboratory animals (NIH, 8023) and the Institutional Animal Care and Use Committee FIOCRUZ (CPqGM 025/2011). Every effort was made to minimize the number of animals used and to avoid any unnecessary discomfort.

### 2.2. Production of Mesenchymal Stem/Stromal Cells (MSC)

MSC were obtained from the bone marrow of femurs and tibiae of male C57Bl/6 mice, following the methods described by Soleimani and Nadri [44]. A population of MSC with spindle-shaped morphology was maintained at 37°C with 5% CO_2_ and expanded during 5-6 passages in DMEM medium supplemented with 2 mM L-glutamine, 1 mM sodium pyruvate, 50 *μ*g/mL gentamycin, and 10% fetal bovine serum (all reagents were acquired from Sigma). When 80-90% confluence was reached, cells were detached using 0.25% trypsin (Invitrogen Molecular Probes, Eugene, OR, USA). Cells were then washed with serum-free medium and resuspended in 0.9% saline solution. Finally, cell suspensions were counted using a hemacytometer and transplanted into mice via tail vein injection (1 × 10^6^ cells/mouse in a final volume of 100 *μ*L of sterile saline solution with 10% heparin).

The identity of MSC was confirmed on the basis of morphological criteria, plastic adherence, and specific surface antigen expression evaluated by flow cytometry. The analysis of cell surface markers showed that MSC expressed low levels of hematopoietic cell lineage markers (0.7% CD45, 1.3% CD34, and 1.6% CD11b) and expressed common MSC-specific cell surface markers such as CD90 (90%), CD44 (99%), CD73 (83%), and Sca-1 (88%). Additionally, the differentiation capacity into osteocytes, adipocytes, and chondrocytes was evaluated after induction using specific media, as previously described [23]. MSC differentiations were confirmed by the presence of mineralizing cells after staining with alizarin red (osteocytes), lipid droplets after staining with oil red (adipocytes), or glycosaminoglycans after staining with Alcian blue (chondrocytes).

### 2.3. OXL-Induced Peripheral Neuropathy Model

Following the methods employed by Azevedo et al. [45], the chronic peripheral neuropathy model in mice was established by intravenous administration of OXL (1 mg/kg), twice a week in alternating days, for 4.5 weeks, totaling 9 administrations. A stock solution of OXL was prepared in distilled water (1 mg/mL). At the time of administration, the stock solution was diluted in 5% dextrose solution (final concentration 0.1 mg/mL). After baseline assessments of motor performance, body weight, and nociceptive thresholds, the animals started the induction cycle by repeated injection of OXL through the lateral vein of the tail. The control group mice received nine intravenous injections of vehicle (5% dextrose) instead of OXL. The OXL-induced chronic painful neuropathy development was evidenced by the reduction of the nociceptive thresholds.

### 2.4. Mechanical and Thermal Nociceptive Threshold Evaluation

Sensory parameters of OIPN were assessed throughout the experimental period by using the established behavioral assays that evaluate mechanical and thermal nociceptive thresholds [46, 47], as described by Evangelista et al. [23], and the method description partly reproduces their wording. Withdrawal threshold to mechanical stimulation was measured using von Frey filaments (Stoelting; Chicago, IL, USA). In a quiet room, mice were placed in acrylic cages (12 × 10 × 17 cm) with a wire grid floor, which allowed full access to the ventral aspect of the hind paws, 40 min before the beginning of the test. A series of 9 filaments (0.04–4 g) was applied to the plantar surface of the ipsilateral hind paw to determine the threshold stiffness required for 50% paw withdrawal, according to the nonparametric method of Dixon, as described by Chaplan et al. [46, 48]. A positive response was characterized by the removal of the paw followed by clear flinching movements. The development of OIPN was characterized by mechanical allodynia, indicated by the reduction of the paw withdrawal threshold (in grams).

Withdrawal threshold to cold stimulation was determined using the cold plate test, following the methods described by Ta et al. [47]. Mice underwent an acclimatization period before the beginning of the test. The mice were placed on a smooth surface at controlled temperature at -2.5°C, remaining for 5 minutes under observation. Total nociceptive behaviors, such as licking, shaking, and raising the hind paw, were recorded. The increase in nociceptive behavior indicates the development of cold thermal hyperalgesia, which is considered a hallmark of the OIPN.

### 2.5. Motor Function Assay

Since the behaviors evaluated in nociceptive tests depend on the integrity of the motor capacity of the mice, the motor performance was monitored throughout the experimental period using the rotarod test. We followed the methods of Santos et al. [49], and the method description partly reproduces their wording. The rotarod apparatus (Insight, Ribeirão Preto, Brazil) consisted of a bar with a diameter of 3 cm, subdivided into five compartments. The bar rotated at a constant speed of 8 revolutions per min. The animals were selected 24 h previously by eliminating those mice that did not remain on the bar for two consecutive periods of 120 s. On the test day, mice from different experimental groups were placed on a rotating rod, and the resistance to falling was measured up to 120 s. Mice treated with diazepam (10 mg/kg; Cristália, Itapira, Brazil), the reference drug of the test, were placed on a rotating rod one hour after treatment. The results are expressed as the average time (s) the animals remained on the rotarod in each group.

### 2.6. Experimental Design

Mice were divided into the following experimental groups (*n* = 6): nonneuropathic mice (received nine intravenous injections of 5% dextrose instead of OXL; control group), neuropathic mice plus vehicle of MSC treatment (OXL+saline), and neuropathic mice plus MSC treatment (OXL+MSC). For the behavioral assays, we also included a gold standard analgesic group and control: neuropathic mice plus vehicle of GBP treatment (OXL+saline) and neuropathic mice plus GBP (OXL+GBP, reference drug). Mechanical and thermal nociceptive thresholds were evaluated at baseline and daily after OIPN induction in all groups. Two weeks following the model induction, and after the establishment of the painful neuropathy evidenced by nociceptive threshold reduction, mice received a single administration of MSC 1 × 10^6^ MSC/mouse in a final volume of 100 *μ*L via tail vein, or saline (100 *μ*L), following the methods described by Evangelista et al. [23]. The number of transplanted MSC was defined based on previous works [23, 26]. The effect of GBP, considered the gold standard in the clinical control of sensory neuropathies, was investigated here in order to validate the predictive capacity of the chronic sensory neuropathy model used and to evidence the magnitude of the MSC-induced antinociceptive effect. To this end, two weeks following the model induction, neuropathic mice received oral administrations of GBP (70 mg/kg; 12/12 h) or vehicle (saline 12/12 h) for six consecutive days. During the 6 days of GBP treatment, nociceptive thresholds were daily assessed in two moments, immediately before and one hour after the first dose of the day, and then, daily until the end of the experimental period. The dose and frequency of GBP administration used here were defined based on a previous work [50]. Nociceptive behavioral tests were performed during the 10-week experimental period, both in MSC-treated and GBP-treated mice. In all experimental groups, motor performance and body weight were recorded weekly.

### 2.7. Morphological and Morphometric Analysis of Mitochondria in Primary Afferent Neurons

Four weeks after MSC transplantation, mice were euthanized and sciatic nerve samples (±1 cm) and DRG (L4-L5) were collected, processed, and subjected to morphological and morphometric analyses by transmission electron microscopy, following the methods described by Evangelista et al. [23]. The samples were fixed in 2.5% glutaraldehyde (grade I, Sigma) in 0.1 M sodium cacodylate buffer overnight; washed in cacodylate buffer; postfixed in 1% osmium tetroxide (Sigma), 0.8% potassium ferricyanide, and 5 mM CaCl_2_ in the same buffer for 60 min; serially dehydrated using graded acetone; and embedded in Poly/Bed resin (Polysciences, Warrington, PA, USA). For ultrastructural analysis of mitochondria in sensory fibers of the sciatic nerve and DRG neurons, ultrathin sections (70 nm) were stained with 5% uranyl acetate for 30 min and 15% lead citrate for 5 min and observed in JEOL electron microscope (JEM-1230). Morphological and morphometric evaluations of mitochondria in myelinated and unmyelinated neurons were performed, following the methods described by Flatters and Bennett [51]. Briefly, we counted 60 myelinated fibers and 60 unmyelinated fibers in each sample (*n* = 6) and quantified the total number of mitochondria in each fiber. Mitochondrial atypia was evidenced by increased organelle size (length greater than 265 nm), formation of vacuoles greater than 50% of the mitochondrial area, and accumulation of electron-dense amorphous material on mitochondrial poles, in line with previously established criteria [51]. The level of mitochondrial atypia was represented as a percentage in relation to the total mitochondrial count. Analyses were performed using the software Image-Pro Plus 7.01 (Media Cybernetics, Rockville, MD, USA).

### 2.8. Real-Time PCR

Four weeks after MSC transplantation, mice were euthanized and sciatic nerve samples (±1 cm) and DRG (L4-L5) were collected and processed for the gene expression analysis by real-time quantitative polymerase chain reaction (qRT-PCR). The transcription of the antioxidant genes superoxide dismutase (*Sod1*) and nuclear factor-2 erythroid related factor-2 (*Nrf2*) was evaluated as described by Santos et al. [52]. RNA was extracted of the sciatic nerve and DRG samples using TRIzol reagent (Invitrogen Molecular Probes, Eugene, OR, USA), and the RNA concentration was determined by photometric measurement (NanoDrop 2000c Spectrophotometer; Thermo Fisher Scientific, Waltham, MA, USA). A High-Capacity cDNA Reverse Transcription Kit (Applied Biosystems, Foster City, CA, USA) was used to synthesize cDNA from 1 *μ*g of RNA, according to the manufacturer's recommendations. The qPCR was prepared with TaqMan® Universal PCR Master Mix (Applied Biosystems). qRT-PCR assays were performed to detect the expression levels of *Sod1* (Assay ID Mm01344233_g1) and *Nrf2* (Assay ID Mm00477784_m1) (Thermo Fisher Scientific). All reactions were run in triplicate on an ABI 7500 Real-Time PCR System (Applied Biosystems) under standard thermal cycling conditions. A nontemplate control (NTC) and nonreverse transcription controls (No-RT) were also included. The samples were normalized with *Gapdh* (Assay ID Mm99999915_g1). The threshold cycle (2-*ΔΔ*Ct) method of comparative PCR was used to analyse the data, as described by Schmittgen and Livak [53].

### 2.9. Estimation of Nitrite and Lipid Peroxidation

To determine the levels of nitrite and lipid peroxidation, we followed the methods described by Evangelista et al. [23], and the description of the methods partially reproduces their wording. Four weeks after MSC transplantation, samples of sciatic nerve (±1 cm) and DRG (L4-L5) were collected bilaterally, rinsed with ice-cold saline and homogenized in chilled phosphate buffer (pH 7.4). The malondialdehyde (MDA) content, a marker of lipid peroxidation, was assayed in the form of thiobarbituric acid-reactive substances, as previously described by Tiwari et al. [54]. Briefly, 0.5 mL of homogenate and 0.5 mL of Tris-HCl were incubated at 37°C for 2 h. After incubation, 1 mL of 10% trichloroacetic acid was added, and the sample was centrifuged at 1000 g for 10 min. To 1 mL of supernatant, 1 mL of 0.67% thiobarbituric acid was added, and the tubes were kept in boiling water for 10 min. After cooling, 1 mL of double distilled water was added, and the absorbance was measured at 532 nm. The total protein concentration of the samples was estimated using the Bradford protein assay, and the malondialdehyde content was expressed as micromoles of malondialdehyde per milligram of protein (*μ*mol/mg). Nitrite was estimated in the sciatic nerve and DRG homogenate using the Griess reagent and was used as an indicator of nitric oxide production [55]. Briefly, 500 *μ*L of Griess reagent (1 : 1 solution of 1% sulfanilamide in 5% phosphoric acid and 0.1% naphthylamine diamine dihydrochloric acid in water) was added to 100 *μ*L of homogenate. Absorbance was measured at 546 nm, and the nitrite concentration, expressed as micrograms of nitrite per milligram of protein (*μ*g/mg), was calculated using a standard curve of sodium nitrite.

### 2.10. Statistical Analyses

All data are presented as means ± SD of measurements made on six animals in each group. For repeated measures (nociceptive threshold data), comparisons between groups were made by two-way ANOVA with Bonferroni's post hoc test. The factors analysed were treatments, time, and treatment-time interaction. Remaining data were analysed using one-way ANOVA followed by Tukey's posttest. For morphometric analysis, Shapiro-Wilk test was performed, and because all data were positive for normality, one-way ANOVA was used. All data were analysed using the GraphPad Prism v.5.0 software (GraphPad, San Diego, CA, USA). Differences were considered statistically significant for *p* values < 0.05.

## 3. Results

### 3.1. MSC Transplantation Reverts Long-Term Sensory Dysfunctions in Mice with OIPN

**Figure 1:**
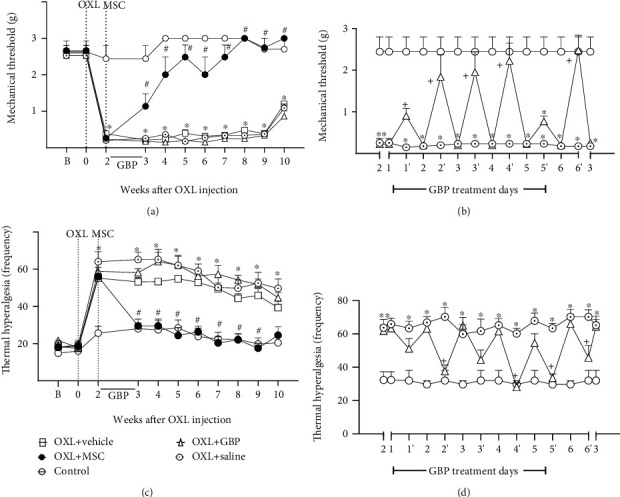
Pain-like behaviors of mice treated with MSC or gabapentin on the OXL-induced painful neuropathy model. (a, b) show mechanical nociceptive threshold data: the strength of the filament (grams) in which the animal responds in 50% of the presentations (*y*-axis) in relation to time (*x*-axis), given in weeks (a) or days (b), after induction of the neuropathy model. (c, d) show cold thermal hyperalgesia data: the frequency of nociceptive behaviors (*y*-axis) in relation to time (*x*-axis), given in weeks (c) and days (d), after OXL induction. The nociceptive threshold was evaluated in the mice before (b) and after OXL induction (week 0). The control group represents nonneuropathic mice that received vehicle (5% dextrose) instead of OXL. Two weeks after the model induction, mice were intravenously treated with MSC (OXL+MSC; 1 × 10^6^/100 *μ*L) or vehicle (OXL+vehicle; 100 *μ*L) or orally treated with gabapentin (OXL+GBP; 70 mg/kg; 12/12 h; 6 consecutive days) or saline (OXL+saline; 12/12 h; 6 consecutive days). During the 6 days of GBP treatment, nociceptive thresholds were daily assessed in two moments: immediately before and one hour after the GBP administration. (b, d) show nociceptive threshold data before (time points 1, 2, 3, 4, 5, and 6) and after (time points 1′, 2′, 3′, 4′, 5′, and 6′) the administration of GBP. Data are expressed as means ± standard deviation; *n* = 6 mice per group. ^∗^Statistical significance relative to the control group (*p* < 0.001); ^#^statistical significance relative to the OXL+vehicle group (*p* < 0.001); ^+^statistical significance relative to the OXL+saline group (*p* < 0.05), as determined by two-way ANOVA followed by Bonferroni posttest.

In the OIPN model, behavioral assessment showed that MSC-treated neuropathic mice have nociceptive thresholds increased for mechanical and thermal stimuli, while GBP induced just transient effects in neuropathic mice. Behavioral signs of painful neuropathy, characterized by the reduction of nociceptive thresholds, were evident starting from the second week of the OXL administration cycle. After the model induction, mechanical allodynia (Figure 1(a)) and cold thermal hyperalgesia (Figure 1(c)) persisted throughout the experimental period (10 weeks; *p* < 0.001). To verify whether MSC were able to revert the established painful neuropathy, mice were treated with MSC (1 × 10^6^, 100 *μ*L) or vehicle (100 *μ*L), 2 weeks after OIPN induction. One week after transplantation, MSC-treated neuropathic mice exhibited reduction of the behavioral painful neuropathy compared to vehicle-treated neuropathic mice (*p* < 0.001). The antinociceptive effect of MSC was progressive, achieving a complete reversal of mechanical allodynia (Figure 1(a); *p* < 0.001) and thermal hyperalgesia (Figure 1(c); *p* < 0.001) in 2 and 1 weeks after treatment, respectively. Over the second week, a group of mice was daily treated with the gold standard drug GBP (70 mg/kg, 12/12 h, p.o.), whereas a control group received saline solution (p.o.). GBP-treated neuropathic mice exhibited a transient antinociceptive effect (Figures 1(b) and 1(d), *p* < 0.05) during the treatment period. There were no signs of motor impairment in mice from the different experimental groups, as assessed by the rotarod test (data not shown).

### 3.2. MSC Treatment Reduces the Frequency of Mitochondrial Atypia in Primary Afferent Neuron Fibers of the Sciatic Nerve of OIPN Mice

**Figure 2:**
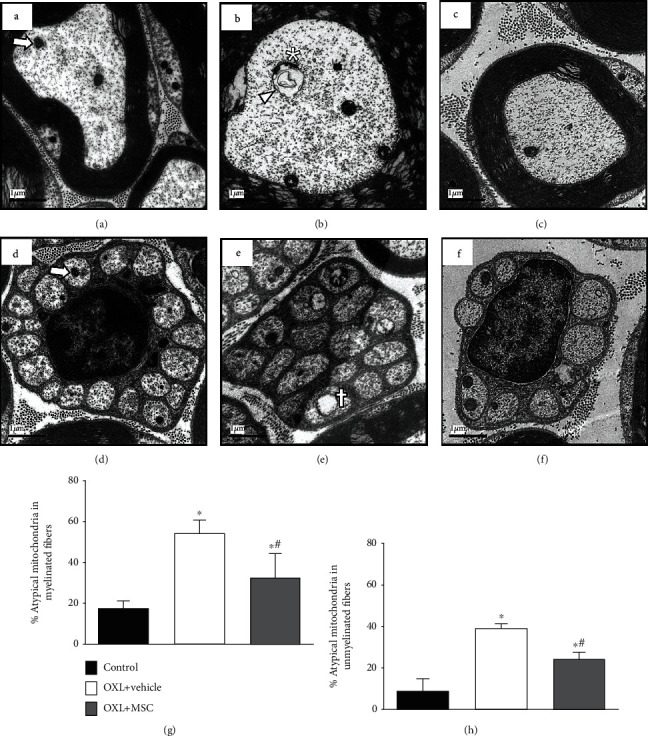
Effect of MSC on mitochondrial morphology and morphometry on sciatic nerve sensory fibers of mice with OXL-induced chronic neuropathy. Transmission electron micrographs of sciatic nerve cross-sections from nonneuropathic mice (control group) and neuropathic mice treated with vehicle (OXL+vehicle) or with 1 × 10^6^ MSC (OXL+MSC). Panels show myelinated (a–c) and unmyelinated (d–f) fibers of the sciatic nerve. Analyses were performed 4 weeks after treatments. Electron micrograph of the control group reveals mitochondria with double intact membrane and absence of vacuolated intramitochondrial spaces and organelles with normal proportion area (arrow) in both myelinated (a) and unmyelinated fibers (d). Vehicle-treated neuropathic mice presented atypical mitochondria, characterized by increased organelle size (arrowhead), presence of vacuole greater than 50% of the mitochondrial area (asterisk), and accumulation of electron-dense amorphous material at the mitochondrial pole (cross), in both myelinated (b) and unmyelinated (e) fibers. MSC-treated neuropathic mice presented fewer mitochondrial morphological alterations in sensory fibers relative to vehicle-treated neuropathic mice (c, f). (g, h) show the percentage of atypical mitochondria found in myelinated and unmyelinated fibers, respectively. Scale bar = 1 *μ*m. Data are expressed as means ± standard deviation; *n* = 6 mice per group. ^∗^Statistical significance relative to the control group (*p* < 0.001); ^#^statistical significance relative to the OXL+vehicle group (*p* < 0.05), as determined by one-way ANOVA followed by Tukey's multiple comparison test.

Since OIPN is associated with mitochondrial alterations in the peripheral nervous system, morphologic and morphometric analyses of mitochondria present in myelinated and unmyelinated fibers of the sciatic nerve were evaluated by transmission electron microscopy. Based on preestablished parameters of mitochondrial atypia, such as size, vacuoles, and swelling, electron photomicrographs of the sciatic nerves of nonneuropathic mice showed a low occurrence of atypical mitochondria in axons of myelinated fibers (Figure 2(a)) and unmyelinated fibers (Figure 2(d)). Mice with OXL-induced chronic neuropathy showed an increased incidence of vacuolated and swollen mitochondria in peripheral nociceptive neurons (Figures 2(b) and 2(e)) compared to control nonneuropathic mice. Morphometric data showed that 54.4% of mitochondria in myelinated fibers (Figure 2(g)) and 39.3% of mitochondria in unmyelinated fibers (Figure 2(h)) were atypical in vehicle-treated neuropathic mice. In nonneuropathic mice, 17.6 and 8.9% of mitochondrial atypia were evidenced in myelinated and unmyelinated fibers, respectively. These data show a relevant increase in mitochondrial atypia in mice with OXL-induced neuropathy (*p* < 0.001). In contrast, MSC-treated neuropathic mice had a reduction (*p* < 0.001) in the proportion of atypical mitochondria in myelinated (Figure 2(c)) and unmyelinated fibers (Figure 2(f)).

**Figure 3:**
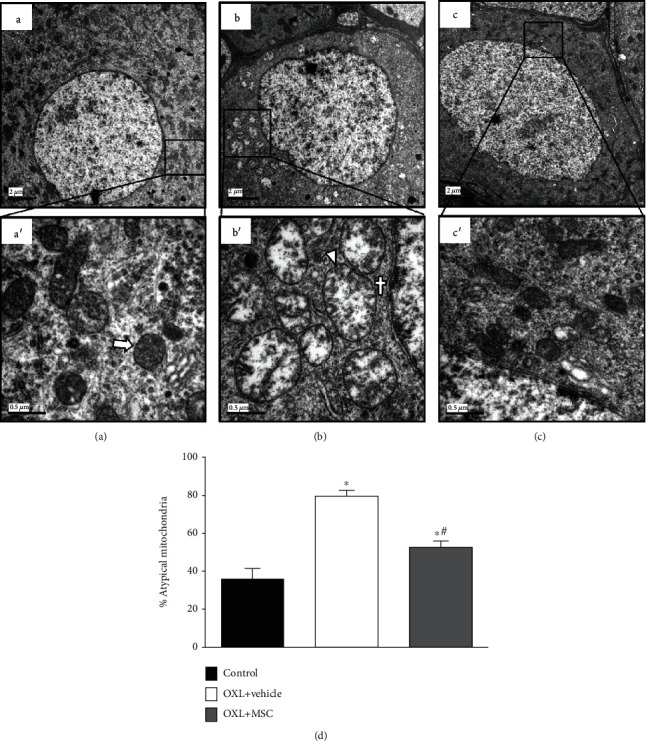
Effect of MSC on mitochondrial morphology and morphometry of dorsal root ganglia neurons of mice with OXL-induced chronic neuropathy. Electron micrographs representing dorsal root ganglia (DRG) cross-sections from nonneuropathic mice (A, D) (control group) and neuropathic mice treated with vehicle (B, E) (OXL+vehicle) or with 1 × 10^6^ MSC (C, F) (OXL+MSC). Analyses were performed 4 weeks after treatments. (A) Electron micrograph of the control group with numerous typical mitochondria. (D) Higher magnification (50,000x) of the highlighted area in (A), showing typical mitochondria, with intact membrane and mitochondria cristae (arrow). (B) Neuropathic mice treated with vehicle presented numerous atypical mitochondria. (E) Higher magnification image showing atypical mitochondria, characterized by increased organelle area (arrowhead) and accumulation of electron-dense amorphous material on mitochondrial poles (cross). MSC-treated neuropathic mice (C, F) presented fewer mitochondrial atypia relative to vehicle-treated neuropathic mice. Scale bar = 2 *μ*m (A–C) (10,000x magnification) and 0.5 *μ*m (D–F) (50,000x magnification). (G) shows the percentage of atypical mitochondria found in DRG. Data are expressed as means ± standard deviation; *n* = 6 mice per group. ^∗^Statistical significance relative to the control group (*p* < 0.001); ^#^statistical significance relative to the OXL+vehicle group (*p* < 0.05), as determined by one-way ANOVA followed by Tukey's multiple comparison test.

Morphologic and morphometric analyses of the DRG (Figure 3) showed a similar profile of mitochondrial atypia to that observed in the sciatic nerve fibers. Neuropathic mice (Figures 3(B) and 3(E)) showed an increased percentage of atypical mitochondria compared to the control group (Figures 3(A) and 3(D); *p* < 0.001). Electron microscopy data showed that nonneuropathic mice had 35.9% of DRG atypical mitochondria, whereas 79.5% were atypical in OXL-neuropathic mice (Figure 3(G)). MSC treatment was able to reduce (*p* < 0.001) the percentage of atypical mitochondria in neuropathic mice (Figures 3(C) and 3(F)).

### 3.3. Treatment with MSC Increased the Antioxidant Profile in the Peripheral Nervous System of Mice with OXL-Induced Chronic Neuropathy

**Figure 4:**
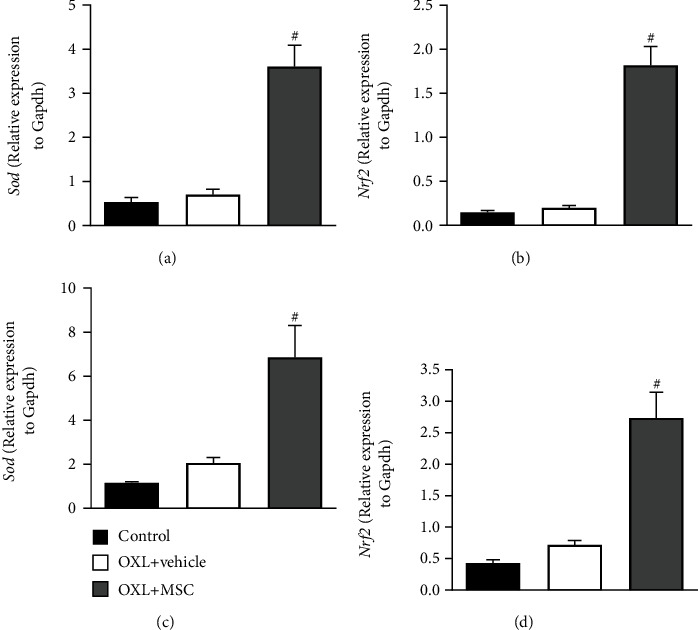
Effect of MSC on the expression of the antioxidant factors, Sod and Nrf2, in the sciatic nerve and dorsal root ganglia of mice with OXL-induced chronic neuropathy. Two weeks after the OIPN model induction, mice were treated with 1 × 10^6^ MSC (OXL+MSC) or vehicle (OXL+saline) by endovenous route. Control group received 5% dextrose instead of OXL. The levels of mRNA in the sciatic nerve (a, b) and DRG (c, d) were measured by RT-qPCR 4 weeks after treatment. Target gene expression is represented normalized by the internal control gene, *Gapdh*. Data are expressed as means ± standard deviation; *n* = 6 mice per group. ^#^Statistical significance relative to the OXL+vehicle group (*p* < 0.01), as determined by one-way ANOVA followed by Tukey's multiple comparison test.

Considering the mitochondrial data and the key role of oxidative stress in the pathophysiology of OIPN, the effect of MSC on the redox balance in the peripheral nervous system of neuropathic mice was next evaluated. Four weeks after transplantation, MSC-treated neuropathic mice presented increased expression of *Sod* and *Nrf2* mRNA in the sciatic nerve (Figures 4(a) and 4(b), respectively) and DRG (Figures 4(c) and 4(d), respectively), when compared to vehicle-treated neuropathic mice (*p* < 0.01). Vehicle-treated neuropathic mice showed similar levels of *Sod* and *Nrf2* mRNA compared to control mice.

### 3.4. Effect of MSC Treatment on Oxidative Stress Markers in the Peripheral Nervous System during OXL-Induced Chronic Neuropathy

**Figure 5:**
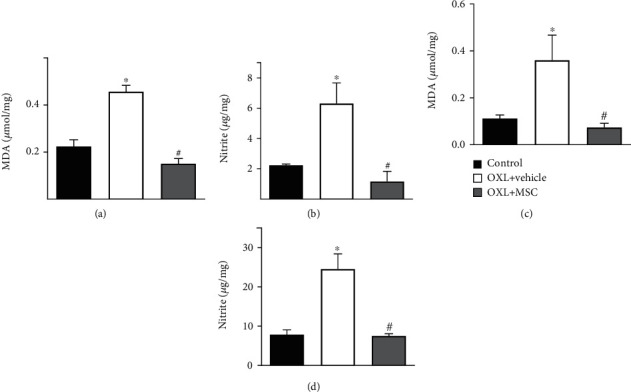
Effect of MSC on the levels of MDA and nitrite in the sciatic nerves and dorsal root ganglia of mice with OXL-induced chronic neuropathy. Two weeks after the OIPN model induction, mice were treated with 1 × 10^6^ MSC (OXL+MSC) or vehicle (OXL+vehicle) by endovenous route. Control group received 5% dextrose instead of OXL. The levels of MDA and nitrite in the sciatic nerve (a, b, respectively) and DRG (c, d, respectively) were measured 4 weeks after treatment. Data are expressed as means ± standard deviation; *n* = 6 mice per group. ^∗^Statistical significance relative to the control group (*p* < 0.01); ^#^statistical significance relative to the OXL+vehicle group (*p* < 0.001), as determined by one-way ANOVA followed by Tukey's multiple comparison test.

The effects of the MSC transplantation on nitrosative stress and lipid peroxidation were evaluated by measuring the nervous tissue levels of nitrite and MDA four weeks after transplantation (Figure 5). MDA levels were enhanced in the sciatic nerve (Figure 5(a)) and DRG (Figure 5(c)) of neuropathic mice, compared to the levels found in control mice (*p* < 0.01). MSC transplantation reduced (*p* < 0.001) the levels of MDA in the sciatic nerve and DRG of neuropathic mice, which presented levels similar to those observed in nonneuropathic control mice. A similar profile effect was observed with nitrite levels, since the elevated levels found in the sciatic nerve (Figure 5(b)) and DRG (Figure 5(d)) of mice with OXL-induced sensory neuropathy (*p* < 0.01) were fully normalized by MSC transplantation (*p* < 0.001).

## 4. Discussion

The present study demonstrated that treatment with MSC reverts the sensory alterations associated with OXL-induced chronic peripheral neuropathy in mice, in parallel to the restoration of oxidative homeostasis and reduction of mitochondrial atypia in the nociceptive primary afferent. Therefore, this study points out a new mechanism possibly involved in the antinociceptive effect induced by MSC during neuropathic conditions and reinforces the therapeutic potential of these cells for the control of chronic painful neuropathies.

OXL-induced chronic peripheral neuropathy is characterized by increased excitability of nociceptive primary afferent neurons, leading to sensory disorders in patients, which persist even after discontinuity of chemotherapy cycles [14, 56, 57]. The neurotoxic effects of OXL lead to the development of dysesthesias and paresthesias of the hands and feet. These symptoms are often triggered by exposure to cold temperatures, which is a hallmark of OXL-induced painful neuropathy [4, 56]. Cold hyperalgesia, characterized by increased response to a cold painful stimulus, and mechanical allodynia, which is the induction of pain by innocuous stimuli, are frequent sensory symptoms found in patients with OXL-induced neuropathy [56]. Therefore, the sensory manifestation development by OIPN mice was compatible with the classic symptoms manifested by patients with OXL-induced neuropathy, suggesting a good clinical equivalence of the experimental model used in the present study. In addition, it is well described that chemotherapy-induced peripheral neuropathy predominantly affects sensory nerves [58], while the involvement of motor or autonomic nerves is rare [59]. In fact, we showed that chronic treatment of mice with OXL induced persistent changes in nociceptive sensitivity without affecting the motor function, as evidenced by the rotarod test.

MSC transplantation completely reverted the behavioral signs of OXL-induced painful neuropathy, an effect which persisted until the end of the experimental period. In fact, the antinociceptive effect of MSC has been demonstrated in different conditions of sensory neuropathy, both in experimental [60] and clinical [61] conditions. Corroborating the present data, the antinociceptive effect of MSC on oxaliplatin-induced neuropathy has recently been described [30, 62]. Importantly, the long-lasting antinociception of MSC seen in the present study differed drastically from the antinociceptive effect induced by repeated gabapentin administration, which only induced antinociception in the first hours after administration. This long-lasting antinociceptive profile suggests that MSC modulate pathophysiological events of OIPN maintenance, rather than exerting an intrinsic analgesic action. Therefore, a key mechanism of the OIPN, which is the redox imbalance in the peripheral nervous system, was investigated here.

Considering that oxaliplatin causes mitochondrial dysfunction in sensory neurons [63], morphological and morphometric aspects of mitochondria were evaluated. Corroborating the study of Xiao and Bennett [64], transmission electron microscopy analysis showed a high percentage of atypical mitochondria in the primary sensory neurons of mice with OIPN. The correlation between mitochondrial changes and platinum compound-induced sensory neuropathy is already well established. Platinum products when binding to mitochondrial DNA form pt-DNA adducts, which cannot be repaired due to the absence of DNA repair systems in the mitochondria. Such pt-DNA adducts block DNA replication and mRNA transcription impairing mitochondrial integrity and favoring disarrangements in phosphorylative activity [65] in sensory neurons. Thus, the platinum compound family interferes with the synthesis of mitochondrial proteins that can generate several abnormalities to this organelle [11]. Importantly, the present study showed that MSC transplantation reduced mitochondrial atypia in primary afferent neurons of mice with OXL-induced neuropathy. Thus, it is possible that the antinociceptive effect induced by MSC during OIPN results in part from its ability to normalize mitochondria function in peripheral nociceptive neurons.

It is well established that the potential of MSC for tissue repair depends on various mechanisms, ranging from the secretion of paracrine factors and extracellular vesicles [66, 67] to the transfer of cellular organelles [68], such as mitochondria. Mitochondrial transfer between MSC and various cell types, including neural cells, has been well documented [36, 37, 69, 70]. It has been proposed that cell damage acts as signaling for the directional transfer of mitochondria from MSC to damaged cells, and this seems to be a key mechanism for tissue repair mediated by these cells [33]. Recently, it has been proposed that this mechanism may be important for the therapeutic effects of MSC during cisplatin-induced neuropathy [29]. This hypothesis was not evaluated in the present study but will be the subject of future investigations.

It has been proposed that sensory alterations associated with neuropathy induced by platinum compounds originate from mitochondrial damage in peripheral sensory neurons, leading to increased production of reactive oxygen species (ROS), oxidative/nitrosative stress, and energy imbalance in these neurons [71, 72]. The peripheral nervous system is more susceptible to oxidative stress due to the high phospholipid content and weak cellular antioxidant defenses and therefore is vulnerable to the mitotoxic effects of ROS induced by OXL [64, 73]. In OIPN mice, in addition to the ultrastructural analysis revealing mitochondrial morphological damage in nociceptive primary afferent, the dearrangement of oxidative machinery in the peripheral nervous system was evidenced by the mismatch between high expression of oxidative stress markers and the mobilization of cellular antioxidant systems. Although the levels of nitrosative species and malondialdehyde in the peripheral nervous system of mice with OIPN were strongly elevated, the cellular antioxidant systems were apparently not activated, as indicated by the low expression of *Sod* and *Nrf2* in the tissue. Activation of cellular antioxidant defenses is fundamental for the maintenance of redox homeostasis [74]. Mitochondria typically produce superoxide as part of the oxidative phosphorylation process, which is converted into hydrogen peroxide via superoxide dismutase and then to a highly toxic hydroxyl radical. Under normal conditions, the level of intracellular ROS is regulated by excessive ROS removal through enzymatic reactions, which convert ROS to harmless nonradicals. However, excessive ROS may accumulate intracellularly in nonphysiological situations of overproduction or impairment of removal. Under oxidative stress conditions, *Nrf2* is activated and neutralizes ROS via upregulation of transcriptional antioxidant enzymes, such as Sod [75].

In this work the increase in nitric oxide levels and lipoperoxidation products was observed, parallel to the low *Sod* and *Nrf2* expression, in peripheral sensory neurons of mice with OIPN, evidencing that antioxidant defenses become inefficient during chronic neuropathy. This imbalance of the redox homeostasis, associated with OXL-induced mitochondrial dysfunction, creates a vicious cycle of energy dysregulation in peripheral sensory neurons, culminating in chronic painful neuropathy. In fact, the association of elevation of MDA and suppression of Nrf2 with OXL-induced peripheral neuropathy was previously proposed by Yang et al. [63], indicating that the Nrf2 signaling system is a protection mechanism against OIPN. Under conditions of persistent stress induced by OXL, DRG cells produce excessive amounts of ROS, which saturate the antioxidant system and therefore cause the failure of the adaptive antioxidant response, inducing oxidative damage to the peripheral nervous system. In this previous work, the authors pointed out that the activation of the Nrf2 system, before the OIPN development or in its early stages, prevents the ROS-induced damage and stops the progression of peripheral neuropathy. A study conducted by Yehia et al. [76] evidenced that this concept is also true in a clinical setting. The prophylactic effect of L-carnosine against the acute OXL neurotoxicity in colorectal cancer patients was investigated. In L-carnosine-treated patients, an improvement in the sensory neuropathy was associated with increased *Nrf2* expression and reduced lipid peroxidation in serum samples. Here, we showed that the transplantation of MSC in OIPN mice also regulated the antioxidant system by increasing the expression of *Sod* and *Nrf2*, reducing the levels of oxidative products in the nervous tissues. The present results highlight the role of MSC in correcting redox imbalances in the peripheral nervous system. It has been described that Nrf2 activation influences mitochondrial biogenesis [77, 78], especially under stress conditions, which may be especially relevant during OIPN, which has OXL-induced mitotoxicity as a central pathophysiological component. Thus, it is possible that the upregulation of *Nrf2* is an important mechanism by which MSC induce a long-lasting antinociceptive effect during OIPN.

## 5. Conclusions

Altogether, these results indicate that the antioxidant properties of MSC mediated by Nrf2, combined with its ability to restore mitochondrial homeostasis, even after prolonged exposure to damage, may contribute to reversal of painful neuropathy induced by OXL. Several lines of evidence have indicated that the antinociceptive effect of MSC during painful neuropathies may depend on multiple mechanisms, which include activation of analgesic endogenous pathways, neuromodulation, immune regulation, and regenerative effects on the peripheral nerve. The present work provides evidence for a new mechanistic hypothesis to explain the long-lasting antinociceptive effects of MSC during chronic painful neuropathies, which is based on the ability of MSC to promote the restoration of mitochondria function and redox homeostasis in the nociceptive primary afferent.

## Data Availability

The behavioral, biochemical, and electron microscopy data used to support the findings of this study are available from the corresponding author upon request.
